# Influence of Particle Size Distribution of Coal Gangue on Performance of Prepared Ceramsite

**DOI:** 10.3390/ma19153212

**Published:** 2026-07-28

**Authors:** Hao Guan, Baoqiang Zhao, Ruidong Guo, Yu Li, Lifeng Sun, Lingdong Zeng, Chaohui Wei

**Affiliations:** 1 State Key Laboratory of Advanced Metallurgy, University of Science and Technology Beijing, Beijing 100083, China; gh1956693259@163.com (H.G.); m202511347@xs.ustb.edu.cn (R.G.); 2 China Energy Construction Coal Refuse Technology (Datong) Co., Ltd., Datong 037008, China; sunlifeng@ceec.net.cn (L.S.); zenglingdong@ceec.net.cn (L.Z.);

**Keywords:** coal gangue ceramsite, grinding process, sintering behavior, mechanical properties

## Abstract

The large-scale accumulation of coal gangue has caused serious environmental problems, and converting it into ceramsite is an important pathway for resource utilization. Grinding is a key step in the preparation of coal gangue ceramsite, but the effect of particle size distribution on heat release and ceramsite performance remains unclear. In this study, coal gangue with a calorific value of 699.77 kcal/kg was ground for 1, 2, 3, and 4 h, respectively, followed by pelletizing and sintering. The different ground powders and sintering ceramsites were investigated using particle size analysis, TG-DSC, and XRD, as well as pore structure and strength tests. The results show that for the Datong coal gangue raw material, grinding parameters and sintering regime adopted in this work, the powder milled for 2 h presents a left-shifted particle size distribution curve with a narrow main peak, particle refinement and a concentrated particle size profile (D50 = 8.498 μm, D90 = 23.941 μm). Combined with TG-DSC, XRD, and pore property test results, the 2 h ground powder delivers the most concentrated heat release during low-temperature combustion. This concentrated heat release is inferred to promote high-temperature mineral phase reconstruction and liquid phase formation, thereby generating a dense ceramsite structure featuring low apparent porosity, high closed porosity and excellent mechanical performance (water absorption: 2.99 ± 0.44%; compressive strength: 15.12 ± 0.43 MPa). When the grinding time is extended to 3 h, the particle size distribution broadens, and both the particle size distribution curve and the DSC curve show shoulder peaks, indicating dispersed heat release. Extending grinding time from 3 h to 4 h appears to induce fine-particle agglomeration with heat release becoming more dispersed and decreasing reaction degree, leading to an uneven temperature distribution and deteriorated ceramsite performance.

## 1. Introduction

As the world’s largest coal producer and consumer, China accounts for more than 50% of global annual coal output [1,2,3]. Coal mining and washing processes generate massive amounts of coal gangue, with China’s annual production ranking first in the world by a substantial margin [4,5,6]. The extensive accumulation of coal gangue not only occupies land resources but also triggers spontaneous combustion, acid mine drainage, and the release of heavy metals, posing severe environmental threats [7,8,9,10]. Therefore, the resource utilization of coal gangue has become a key issue for the green and sustainable development of the coal industry.

Coal gangue ceramsite is a lightweight ceramic particle produced by crushing, grinding, pelletizing, and high-temperature sintering using coal gangue as the main raw material. It offers advantages such as low density, high strength, thermal insulation, and chemical corrosion resistance, and is widely used in lightweight concrete aggregates, wastewater treatment filter media, and horticultural substrates [11,12,13,14]. Converting coal gangue into high-performance ceramsite is an important pathway for its high-value utilization and for reducing pollution from stockpiles.

Grinding is a key pretreatment step in the preparation of coal gangue ceramsite. Its function is to refine particles and increase specific surface area through mechanical force, thereby promoting solid-state reactions and densification during subsequent sintering [15,16]. The traditional view is that longer grinding time produces finer powder and better ceramsite performance. However, recent studies have shown that excessive grinding may cause particle size broadening and fine-particle agglomeration, adversely affecting material properties [17]. In the fields of cermets and powder metallurgy, the non-monotonic effects of grinding time on powder characteristics and sintering behavior have been systematically investigated [18,19]. For instance, Zhang et al. [20] reported that in Mo_2_NiB_2_-Ni cermets, particle size continuously decreases with increasing grinding time but tends to saturate beyond 11 h, with optimal mechanical properties achieved at this grinding time; both insufficient grinding and excessive grinding lead to performance degradation. Similarly, Zhang et al. [21] found that Fe–Mn–Si powders exhibit rapid particle size reduction at the initial grinding stage, but prolonged grinding causes agglomeration and particle size rebound, with the optimal grinding window varying with alloy composition. Studies on low-quality coal gangue have also revealed that an optimal grinding window exists: Cheng et al. [22] reported that grinding for 60 min followed by calcination at 650 °C gives an activity index of 80%, while further grinding leads to secondary agglomeration. Nevertheless, for the compositionally complex coal gangue system, how particle size distribution affects heat release and subsequent sintering densification remains insufficiently understood.

Coal gangue is a complex mineral assemblage mainly composed of kaolinite, quartz, calcite, and a small number of carbonaceous components [23,24,25]. Its sintering process generally involves two mutually interacting key stages: the low-temperature combustion stage, during which fixed carbon and volatile components undergo oxidation combustion, releasing gases and generating localized thermal fields [26], and the high-temperature sintering stage, during which aluminosilicate reconstruction occurs with the precipitation of crystalline phases such as mullite and anorthite, accompanied by liquid phase formation and pore filling [27]. The coupling between these two stages determines the final structure and properties of the ceramsite. The synchronicity of combustion directly affects the temperature distribution and pore uniformity of the green body, thereby controlling the efficiency of liquid phase formation and the extent of crystalline phase development [28,29]. Unlike single-inorganic-powder systems, the combustion reaction of carbonaceous components in coal gangue during the low-temperature stage is highly sensitive to the dispersion state of the powder. Excessive grinding may induce fine-particle agglomeration, which could disrupt combustion synchronicity and consequently inhibit densification at the high-temperature stage. This mechanism, however, has not yet been clearly elucidated for the coal gangue system, and the systematic correlation between particle size evolution and the coupled combustion–sintering process, as well as its impact on ceramsite performance, remains insufficiently studied.

To fill this research gap, this study investigates how grinding-induced particle size distribution influences the thermal behavior, phase evolution, pore structure, and mechanical properties of coal gangue ceramsite. The novelty of this work lies in revealing the non-monotonic effect of particle size distribution on ceramsite properties, and elucidating how excessive grinding-induced particle size broadening and agglomeration disrupt combustion synchronicity in the coupled combustion–sintering process and hinder sintering densification, thereby leading to performance degradation. The critical particle size distribution window for preparing high-performance ceramsite with the Datong coal gangue is accordingly identified, providing a theoretical basis and practical process reference for the regulation of raw material pretreatment and the clean resource utilization of coal gangue solid waste.

## 2. Materials and Methods

### 2.1. Coal Gangue

The coal gangue used in this study was obtained from the Tashan mining area in Datong, Shanxi Province, China. Bulk coal gangue was crushed using a jaw crusher (EP-100X60, Hebi Metallurgical Machinery Equipment Co., Ltd., Hebi, China) to pass through a 10 mm sieve, and then dried at 100 °C for 24 h in an air oven to remove free moisture.

**Figure 1:**
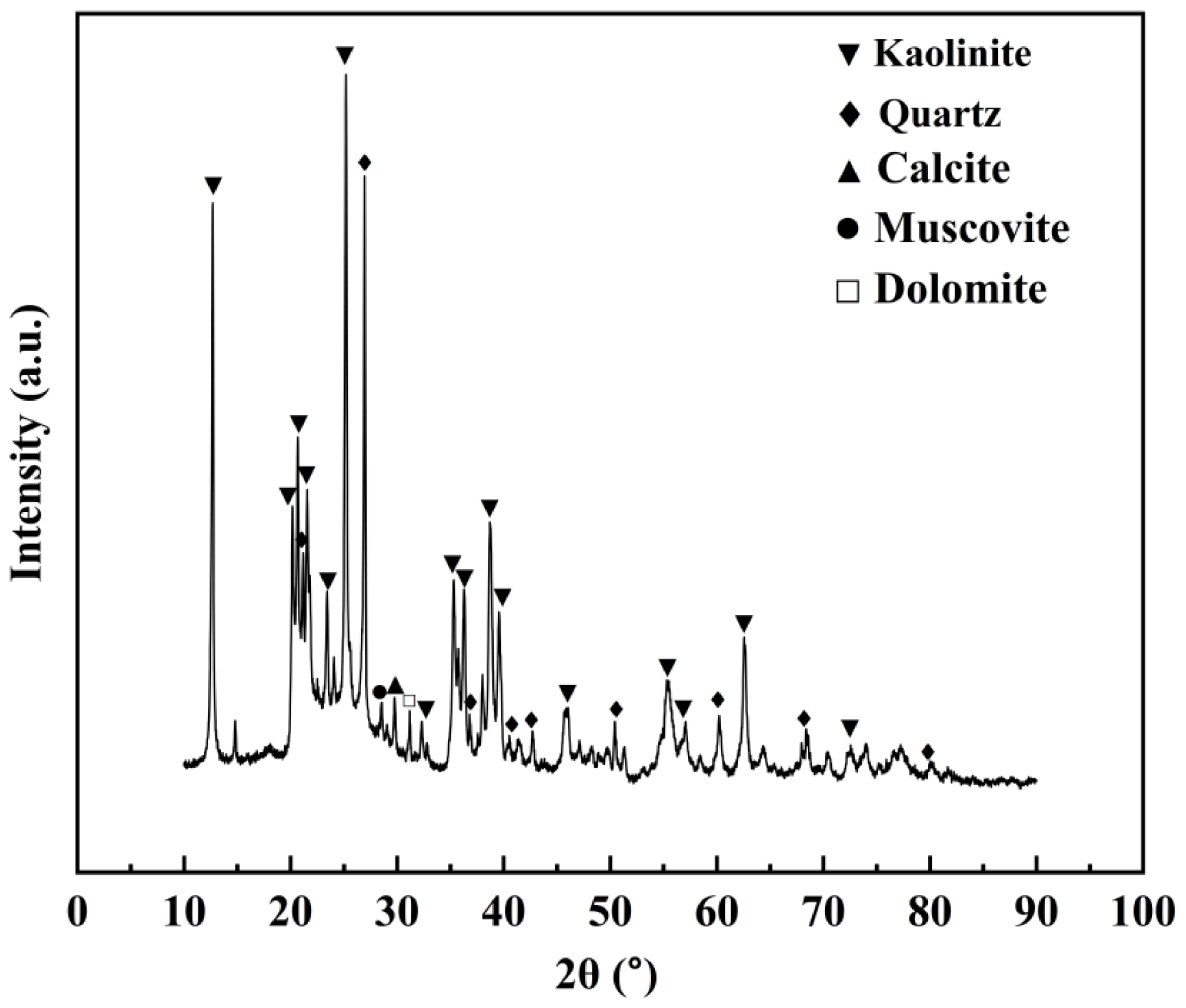
Main mineral phases of coal gangue raw material.

Figure 1 shows the XRD pattern of the raw coal gangue. Its main mineral phases are kaolinite, quartz, and calcite, with small amounts of muscovite and dolomite. The XRF analysis results in Table 1 show that the main chemical components of the raw material are SiO_2_ and Al_2_O_3_, with their combined content exceeding 62.89 wt%; this classifies it as a typical high-silica, high-alumina coal gangue. The loss on ignition is relatively high, primarily due to the removal of carbonaceous components and interlayer water in kaolinite. The industrial analysis and calorific value data in Table 2 indicate that this coal gangue contains a certain amount of volatile matter and fixed carbon.

### 2.2. Grinding and Sintering

The pretreated coal gangue feedstock was subjected to dry grinding in a ball mill (XMQ-420×600, Shicheng Guobang Mining Machinery Co., Ltd., Ganzhou, China). The high-chromium cast iron balls with graded diameters of 40 mm, 50 mm and 60 mm were used as grinding media at a ball-to-powder mass ratio of 10:1. The mill was operated at a constant rotation speed of 48 r/min, with the total filling degree of grinding balls and raw powder controlled at 40% of the jar volume. Each independent grinding batch contained 5 kg of coal gangue and ran continuously for the prescribed duration. By setting the total grinding time to 1 h, 2 h, 3 h, and 4 h, respectively, four powder samples with distinct particle size distributions were prepared for subsequent characterization and sintering tests.

**Figure 2:**
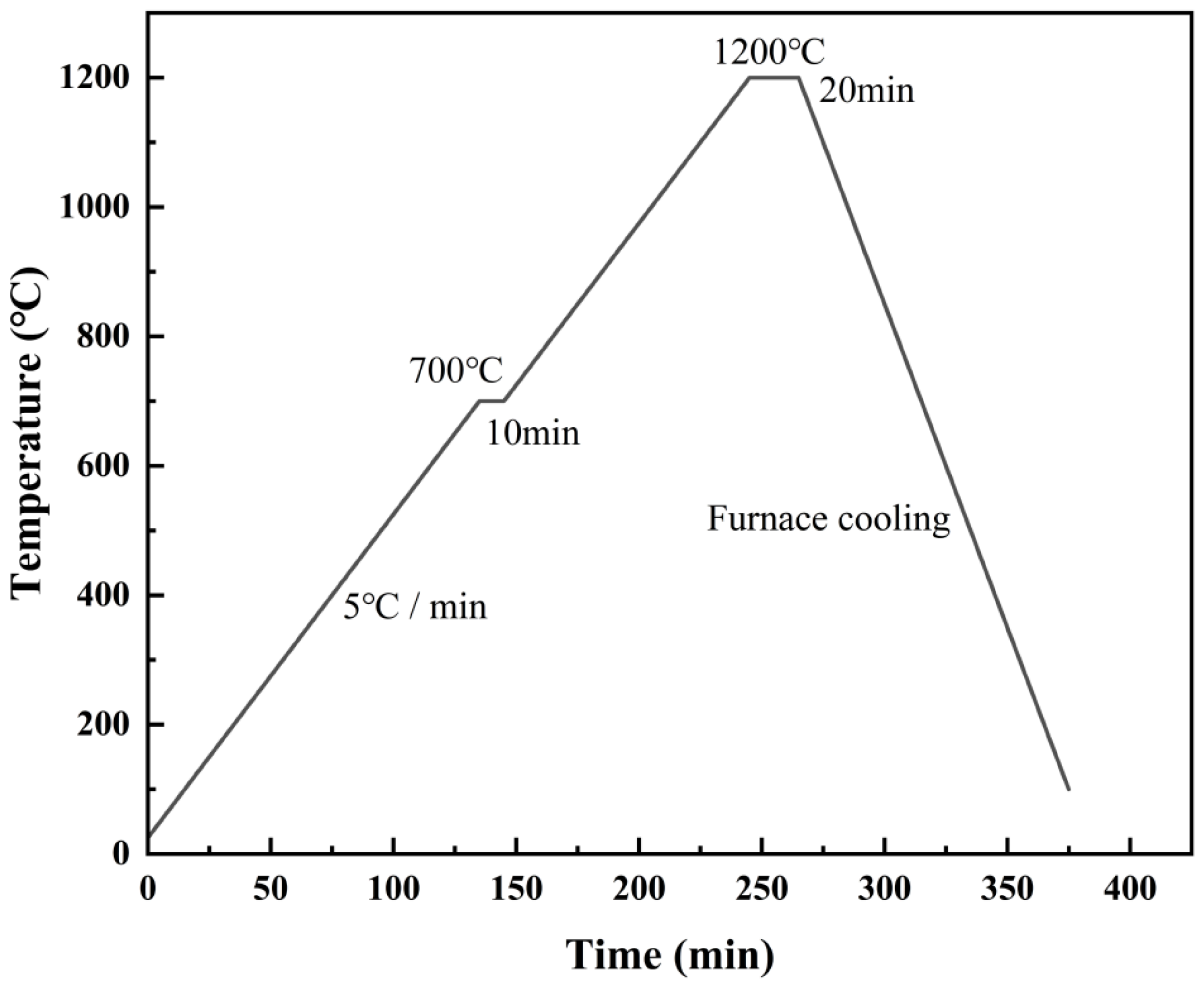
Calcination schedule of coal gangue ceramsite.

Powder samples were pelleted using a rotary granulator at a speed of 50 r/min. No binder was added; water was the only pelletizing liquid. During granulation, water was sprayed into the powder mixture while the granulator was rotating. After granulation, the moisture content of the green pellets was measured using a moisture analyzer (VM-E10, Taizhou Vicometer Instrument Co., Ltd., Taizhou, China) and controlled at 11–13%. The green pellets were further screened to a particle size between 8 and 16 mm. After drying at 100 °C for 2 h, the green pellets were sintered in a muffle furnace (MFLX322-14, Shanghai Muffle Furnace Technology Instrument Co., Ltd., Shanghai, China). The sintering profile is shown in Figure 2: the temperature was raised at a constant rate of 5 °C/min to 700 °C, held for 10 min, then raised to 1200 °C, and held for 20 min, followed by furnace cooling. The resulting ceramsite samples were designated as S1, S2, S3, and S4, corresponding to the powders ground for 1, 2, 3, and 4 h, respectively.

### 2.3. Testing

#### 2.3.1. Characterization

Particle size distribution, median diameter (D50), and specific surface area were measured using a laser particle size analyzer (LS-909, Omec Instruments, Zhuhai, China).

X-ray diffraction (XRD, Ultimate IV, Rigaku, Tokyo, Japan) was employed to identify the phase composition of the powders and ceramsite samples. The operating conditions were Cu Kα radiation (40 kV, 40 mA), scanning speed of 10°/min, and 2θ range of 5–90°. Rietveld refinement [30] was adopted to quantify the phase composition of the samples. The initial crystallographic structure models of quartz, mullite, and anorthite were retrieved from the Inorganic Crystal Structure Database (ICSD). During the refinement process, background function, peak profile parameters, lattice constants and preferred orientation factors were optimized step by step. For all ceramsite samples, the weighted profile R-factor (Rwp) is lower than 14%, and the goodness of fit (χ^2^) ranges from 1.12 to 1.68. The quantitative uncertainty of this method for the present system is ±1 to 3 percentage points.

The chemical composition of the raw coal gangue was analyzed by X-ray fluorescence spectrometry (XRF, ZSX Primus III+, Rigaku, Tokyo, Japan).

Thermogravimetry–differential scanning calorimetry (TG-DSC, HCT-4, Beijing Hengjiu Experimental Equipment Co., Ltd., Beijing, China) was used to investigate the effect of particle size on the sintering behavior of coal gangue. Approximately 20 mg of each sample was placed in a corundum crucible (5 mm outer diameter, 4 mm height) and heated from room temperature to 1100 °Cat 5 °C/min under an air atmosphere (flow rate: 50 mL/min each). TG, DTG, and DSC curves were recorded simultaneously. The DSC peak areas (A1 for the low-temperature stage and A2 for the high-temperature stage) were integrated using the TA Universal Analysis 2000 (Version 4.5A). A linear baseline connecting the onset and end points of each peak was used for integration. The integration ranges were 300–650 °C for A1 and 940–980 °C for A2, based on the temperature intervals where the respective exothermic signals were observed.

#### 2.3.2. Performance Testing

Performance testing of coal gangue ceramsites was conducted in accordance with the Chinese national standards GB/T 17431.1-2010 [31] and GB/T 17431.2-2010 [32]. GB/T 17431.1-2010 specifies the classification and technical requirements of lightweight aggregates, and GB/T 17431.2-2010 provides standardized test methods for lightweight aggregate performance. All physical performance tests (compressive strength, water absorption, total porosity, apparent porosity, and closed porosity) were performed in triplicate (n = 3), and the results are expressed as mean ± standard deviation (SD). One-way analysis of variance (ANOVA) followed by Tukey’s post hoc test was used to evaluate the statistical significance of inter-group differences, with *p* < 0.05 set as the significance threshold.

(1) Compressive strength

The compressive strength of individual ceramsite particles was measured using a ceramic compression testing machine (YDW-5000, Shandong Di’erben Machinery Technology Co., Ltd., Jinan, China) at a loading rate of 2 mm/min. The compressive strength was calculated using Equation (1):

(1) $$F=\frac{2.8P}{\pi d^{2}}$$

*F*—compressive strength of the ceramsite, MPa; *P*—fracture load of ceramsite, N; and *d*—diameter of ceramsite, m.

(2) Water absorption and porosity

Bulk density, apparent porosity, true porosity, closed porosity, and water absorption were determined using a ceramic density analyzer (DT, WKT-300C, Jiangsu Weike Meituo Co., Ltd., Taizhou, China). Bulk density and water absorption were directly measured, while the porosity parameters were calculated using Equations (2)–(5):

(2) $$P_{a}=\frac{M_{3}-M_{1}}{M_{3}-M_{2}}\times 100\%$$

(3) $$D_{b}=\frac{M_{1}D_{1}}{M_{3}-M_{2}}$$

(4) $$P_{t}=\frac{D_{t}-D_{b}}{D_{t}}\times 100\%$$

(5) $$P_{t}=P_{t}-P_{a}$$

*M*_1_—dry weight of the sample, g; *M*_2_—weight of the sample suspended in water, g; *M*_3_—weight of the sample after saturated water adsorption, g; *P_a_*—apparent porosity of the sample, %; *P_c_*—closed porosity of the sample, %; *P_t_*—true porosity of the sample, %; *D_b_*—volume density of the sample, g/cm^3^; *D_t_*—true density of the sample, g/cm^3^; and *D*_1_—density of the immersion liquid, g/cm^3^. The immersion liquid is typically water (density 1 g/cm^3^).

## 3. Results and Discussion

### 3.1. Particle Size Distribution and Powder Characteristics

**Figure 3:**
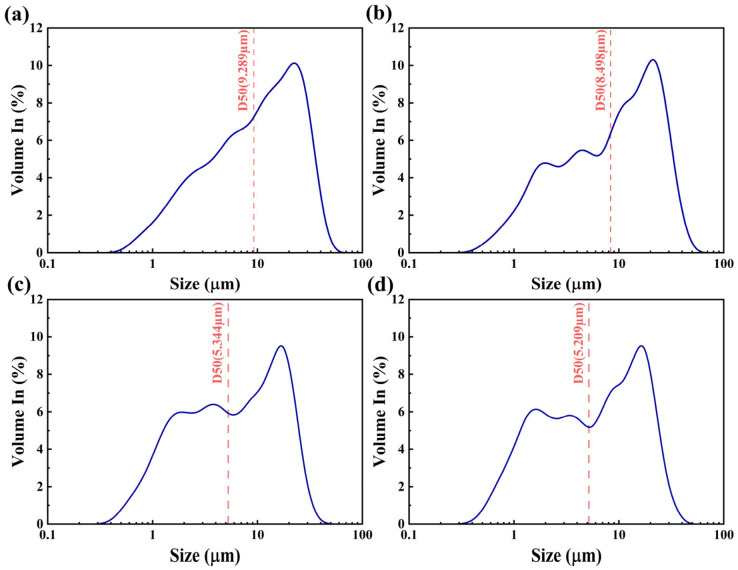
Particle size distributions of coal gangue powders after dry ball milling for different durations: (**a**) 1 h grinding; (**b**) 2 h grinding; (**c**) 3 h grinding; (**d**) 4 h grinding.

**Figure 4:**
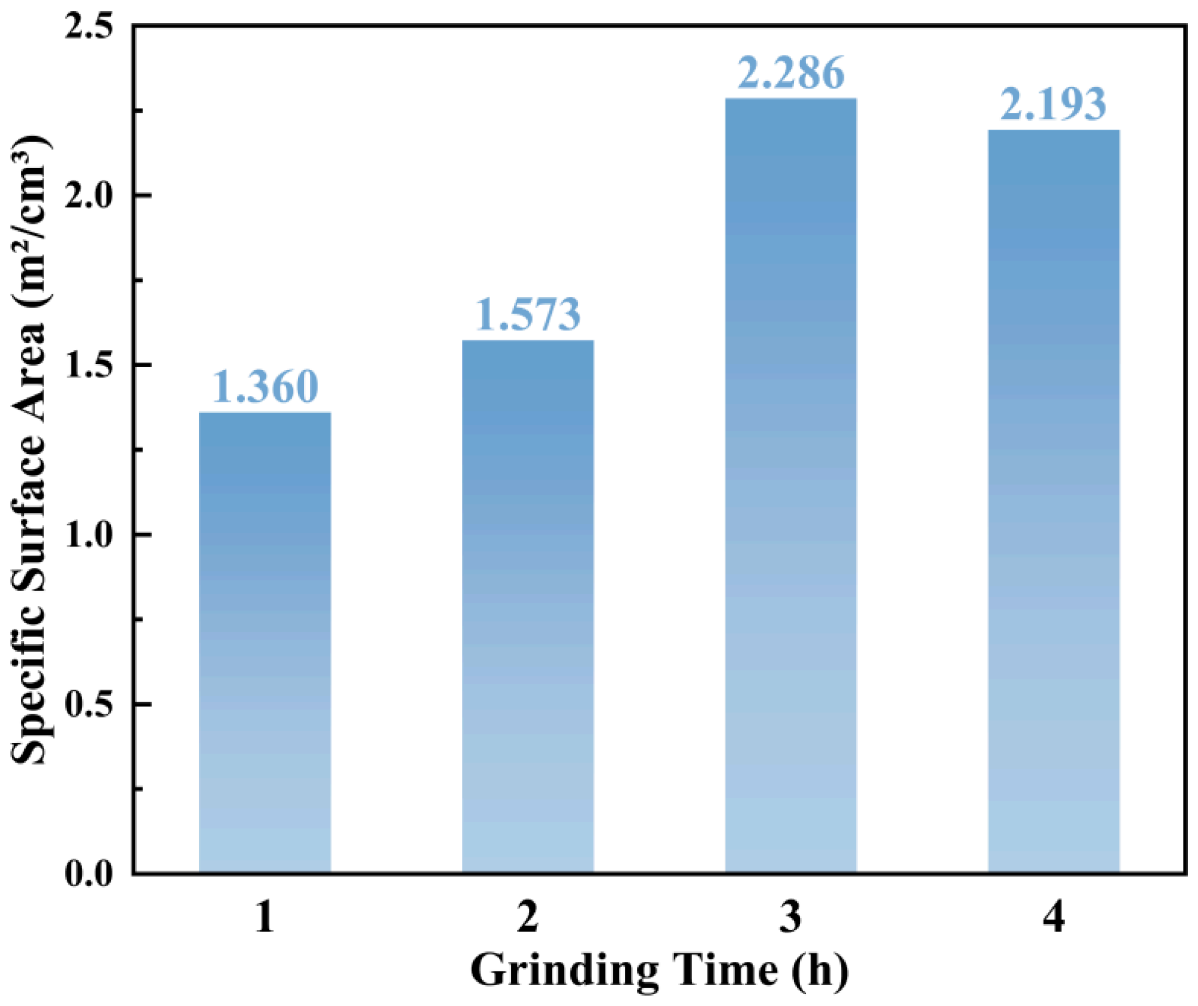
Variation in specific surface area with grinding time.

Figure 3 shows the particle size distributions of coal gangue powders after different grinding times, and Figure 4 presents the corresponding specific surface area. Table 3 summarizes the key particle size parameters, including D10, D50, D90, span, and the volume fractions of four typical particle size intervals, which quantitatively reflect the proportion of particles distributed in different size ranges.

As the grinding time increased from 1 h to 3 h, the particle size distribution curves shifted progressively leftward, accompanied by changes in the volume fraction of each size interval. D50 decreased from 9.289 μm to 8.498 μm after 2 h of grinding, and then to 5.344 μm after 3 h. Meanwhile, the proportion of ultra-fine particles below 2 μm rose continuously from 13.38% to 28.33%, while the volume fraction of coarse particles larger than 22 μm dropped from 15.77% to only 3.20%. The span, defined as (D90 − D10)/D50, increased from 2.564 to 3.073 after 3 h, indicating that the particle size distribution broadened notably. Correspondingly, the specific surface area increased from 1.360 m^2^/cm^3^ to the maximum value of 2.286 m^2^/cm^3^ at 3 h.

When grinding time was extended to 4 h, D_50_ decreased to 5.209 μm, D_10_ increased from 1.025 to 1.087 μm, D_90_ increased from 17.448 to 17.711 μm, and span further rose to 3.191. Meanwhile, the volume fraction of <2 μm ultra-fine particles declined from 28.33% to 26.48%, while the proportion of medium-sized particles of 2–11 μm increased from 42.77% to 44.30%, accompanied by a drop in specific surface area. These changes are consistent with what is typically observed during fine-particle agglomeration in dry ball milling [33].

It should be noted that SEM observation of the ground powders was not included in this study. The conclusion regarding agglomeration is therefore based on multiple consistent indirect indicators from particle size distribution characteristic diameters, sectional volume fractions, specific surface area measurements, and thermal behavior, which collectively present a consistent interpretation.

The effect of grinding time on powder characteristics can be divided into two stages. Within 2 h, particle refinement dominates and the powder remains well dispersed. After 2 h of grinding, the particle size distribution is well concentrated, with a moderate proportion of fine and medium particles, which is conducive to combustion synchronicity. At 3 h, the content of ultra-fine particles reaches the peak, the particle size distribution broadens notably, and the kinetic differences among particles of varying sizes may lead to dispersed heat release. From 3 h to 4 h, the reduction in ultra-fine-particle proportion and specific surface area alongside negligible variation in D50 suggests that agglomeration intensifies, which may further disturb the combustion behavior.

### 3.2. Thermal Behavior of Powders

**Figure 5:**
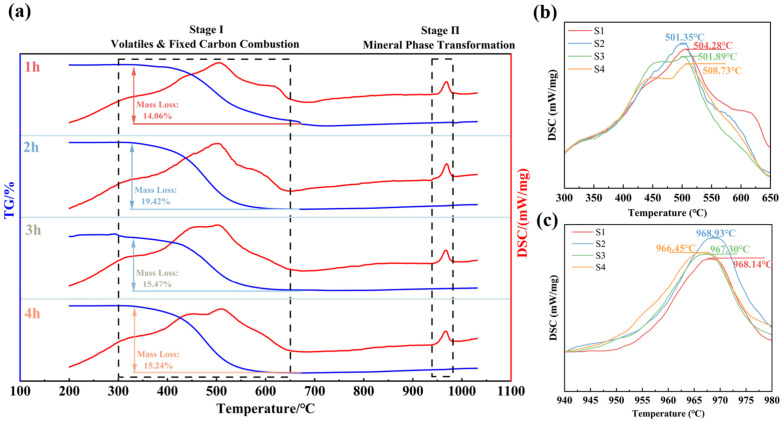
TG-DSC curves of coal gangue powders with different grinding times. (**a**) Full curves from 200 to 1000 °C; (**b**) enlarged DSC curves of the low-temperature stage (300–650 °C); (**c**) enlarged DSC curves of the high-temperature stage (940–980 °C).

**Figure 6:**
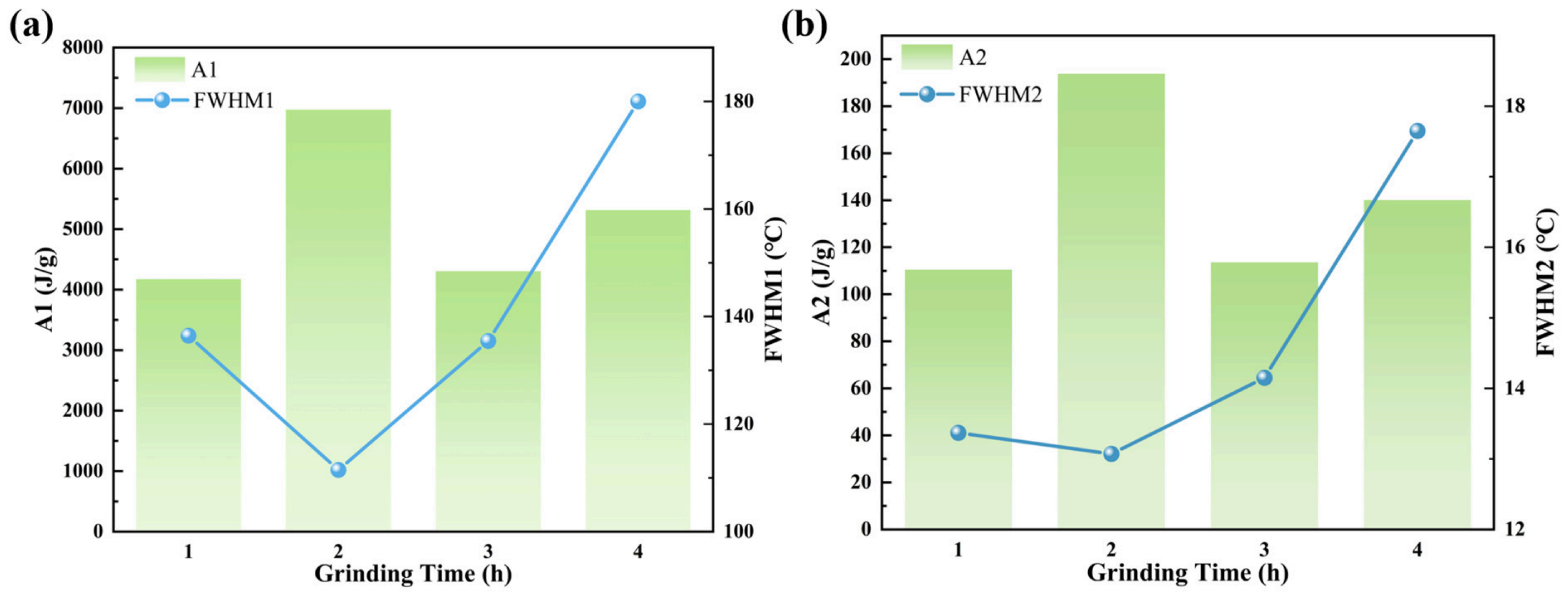
DSC peak parameters for samples with different grinding times. (**a**) Low-temperature stage: A1, peak area; FWHM1, full width at half maximum. (**b**) High-temperature stage: A2, peak area; FWHM2, full width at half maximum.

Figure 5 shows the TG-DSC curves of coal gangue powders with different grinding times. All samples exhibit two exothermic stages. The low-temperature stage (300–650 °C) corresponds to the combustion of fixed carbon and volatiles, which is the primary heat source during ceramsite sintering and directly affects temperature distribution and pore uniformity [26]. The thermal signal in the high-temperature range of 940–980 °C arises from the comprehensive effect of multiple overlapping reactions, including aluminosilicate structural reconstruction, precipitation of crystalline phases such as mullite and anorthite, and liquid phase formation, which jointly determine the densification process and final mechanical properties of the ceramsite [27,34,35]. Figure 6 summarizes the DSC peak parameters of both stages: for the low-temperature combustion stage, peak area (A1) and full width at half maximum (FWHM1) represent the total heat release and its concentration; for the high-temperature mineral phase transformation stage, peak area (A2) and full width at half maximum (FWHM2) represent the enthalpy change and the temperature range of the reaction.

In the low-temperature combustion stage, the 1 h powder shows poor combustion efficiency (A1: 4174 J/g, FWHM1: 136.45 °C) due to its coarse particles and small specific surface area, which limit contact between carbon and oxygen. The 2 h powder shows the highest mass loss, the lowest DSC peak temperature, the narrowest FWHM1 (111.48 °C), and the largest A1 (6941 J/g) (Figure 5b and Figure 6a), indicating that the 2 h powder releases the most heat within the narrowest temperature range. The 3 h powder shows a broader FWHM1 and an asymmetric DSC peak with a shoulder. As shown in Section 3.1, the particle size distribution of the 3 h powder broadens significantly. This shoulder is attributed to the kinetic differences among particles of varying sizes [36]: finer particles combust earlier while coarser particles combust later, spreading heat release over a wider temperature range and reducing the total heat release compared with the 2 h sample. The 4 h powder shows a further broadened FWHM1 and a more pronounced shoulder. From 3 h to 4 h, the specific surface area decreases from 2.286 to 2.193 m^2^/cm^3^ with little change in D50, suggesting the occurrence of agglomeration. This would hinder oxygen penetration into the agglomerates: carbon on the surface burns first, while carbon inside burns later, further dispersing heat release [37].

In the high-temperature mineral phase transformation stage, the DSC peak temperatures of all samples are similar (Figure 5c), but A2 and FWHM2 differ markedly (Figure 6b). The 2 h powder possesses the largest A2 (193.8 J/g) and the narrowest FWHM2 (13.07 °C), indicating that the solid-state reconstruction and crystalline phase precipitation of aluminosilicates are completed efficiently within a narrow temperature range, achieving the highest extent of phase transformation. This is consistent with the low-temperature combustion characteristics: more concentrated heat release within the green body promotes solid-state atomic diffusion and uniform liquid phase formation, thereby facilitating synchronized phase transformation. The 1 h powder shows the weakest mineral reconstruction due to its coarse particles and insufficient solid-state reactivity. For the 3 h powder, the broadened particle size distribution leads to dispersed heat release in the low-temperature stage, which would create a temperature gradient within the green body and inconsistent initiation of solid-state reactions in different regions, resulting in decreased A2 and a broadened reaction temperature range.

The 4 h powder shows a slight increase in A2, but its FWHM2 reaches the maximum among all groups, indicating that the combustion disorder attributed to agglomeration further extends to the high-temperature sintering stage, causing the mineral phase transformation to occur over the widest temperature range with the poorest reaction synchronicity.

Based on the TG-DSC curves, the 2 h ground powder delivers concentrated heat release within a narrow temperature window; this thermal feature is inferred to create relatively homogeneous thermal conditions inside green pellets and may support synchronous, efficient mineral phase transformation into mullite and anorthite. Insufficient grinding results in coarse particles and low reactivity, suppressing both combustion and mineral phase transformation. Grinding for 3 h broadens the particle size distribution, disperses the combustion heat release sequence, and consequently weakens the synchronicity of high-temperature mineral phase transformation. Further extending the grinding to 4 h intensifies agglomeration, which further disrupts the oxidation combustion process and continuously increases the dispersion of mineral solid-state reconstruction, a trend that is detrimental to the sintering preparation of high-performance ceramsite.

### 3.3. Mineral Phase Evolution of Ceramsite

**Figure 7:**
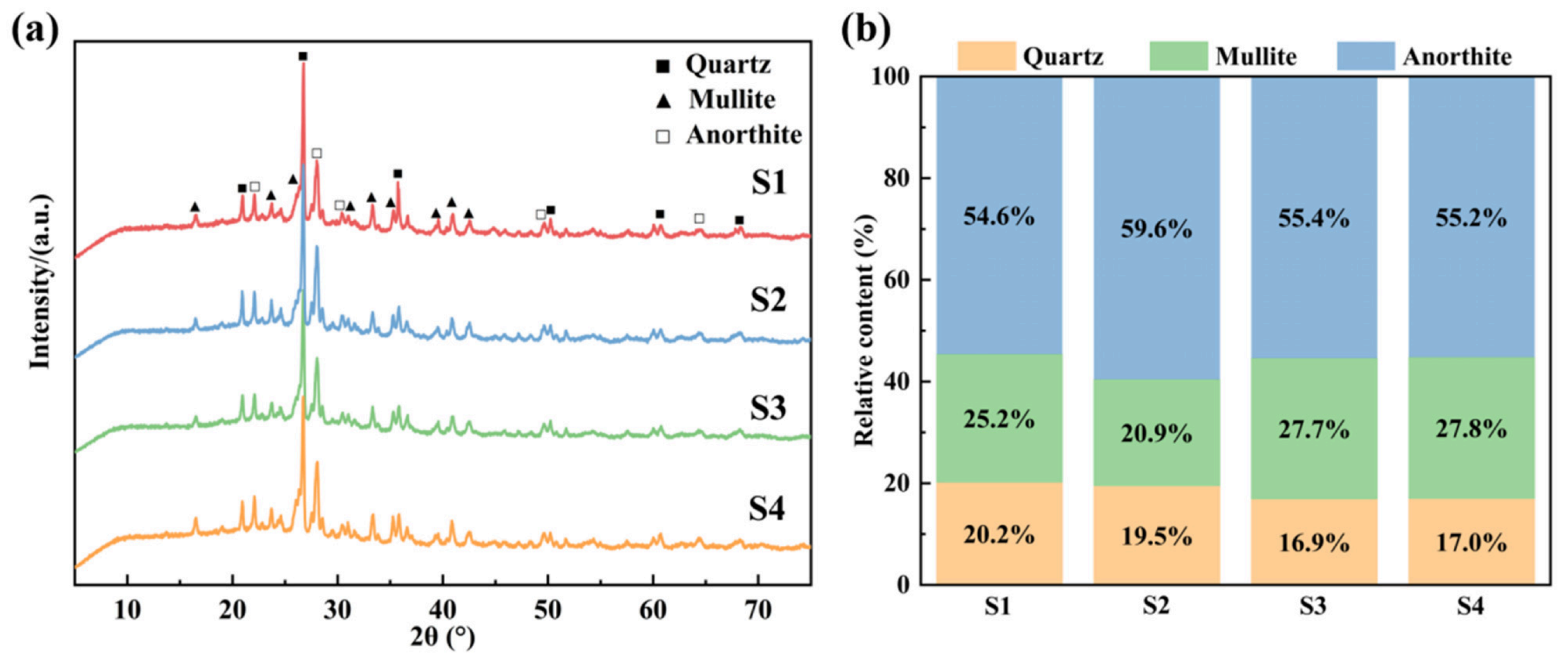
(**a**) XRD patterns of coal gangue ceramsite sintered at 1200 °C with different grinding times and (**b**) quantitative phase contents of quartz, mullite, and anorthite determined by the Rietveld refinement method.

Figure 7a shows the XRD patterns of ceramsite samples sintered at 1200 °C from powders with different grinding times. All samples contain three main crystalline phases: quartz, mullite, and anorthite. Quantitative analysis of the crystalline phase contents was performed using the Rietveld refinement method, and the results are presented in Figure 7b. Mullite provides the rigid skeletal framework of the ceramsite, while anorthite fills the interstices of this skeleton together with the glassy phase. This structure enhances the rigidity and compressive strength of the ceramsite [38].

S1 shows the highest quartz content and the lowest anorthite content. This is attributed to its coarse particles and low specific surface area, which hinder the solid-state reaction from kaolinite to mullite, leaving part of the raw material unreacted.

S2 exhibits the highest anorthite content at 59.6%, with the lowest quartz content at 19.5%. The 2 h powder has a concentrated particle size distribution and achieves more complete combustion with more intensive heat release. Combined with TG-DSC data, this concentrated heat release is speculated to generate more consistent thermal conditions inside pellets and provide adequate driving force for solid-state reactions, which may favor abundant anorthite formation.

S3 shows a lower anorthite content than S2, with higher mullite content and lower quartz content. The broadened particle size distribution leads to dispersed combustion heat release in the low-temperature stage, reducing the synchronicity of the subsequent high-temperature mineral phase transformation. In Si-Al-Ca systems, anorthite is generated via both solid-state reactions among mullite, quartz and other components and liquid-phase-assisted reaction at elevated temperatures [39]. However, the potential premature liquid phase generated under scattered heat release may wrap unreacted particles, hinder atomic diffusion during solid-state reconstruction, and restrict adequate anorthite formation. Consequently, anorthite content decreases while more mullite is retained.

Compared to S3, S4 exhibits a further decrease in anorthite content and the highest mullite content among all samples. Based on the particle size and specific surface area data presented in Section 3.1, this phase assemblage is reasonably interpreted as a consequence of agglomeration. Under such inferred agglomerated conditions, oxygen penetration would be hindered, resulting in delayed combustion inside the agglomerates and less uniform thermal histories across different regions. These effects would in turn suppress the formation of anorthite and favor the retention of mullite.

### 3.4. Pore Structure and Physical Properties of Ceramsite

**Figure 8:**
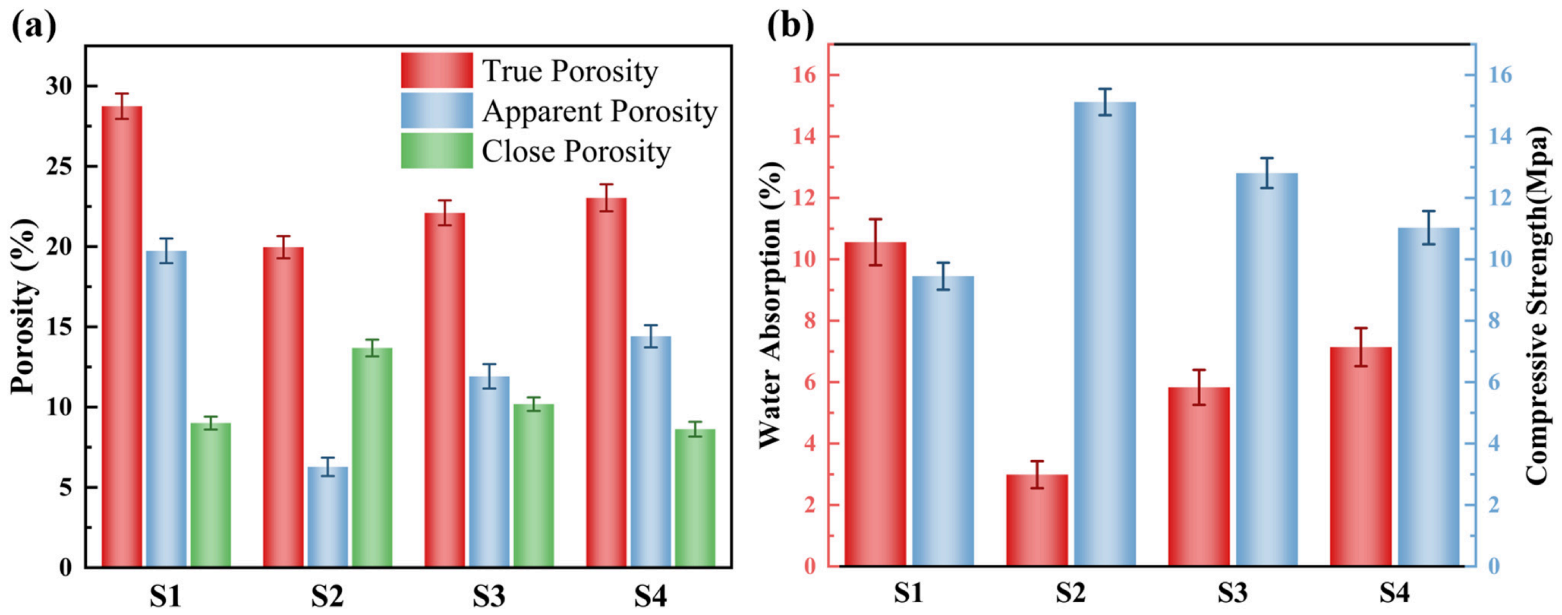
(**a**) True porosity, apparent porosity, and closed porosity of ceramsite samples with different grinding times. (**b**) Water absorption and compressive strength. Data are presented as mean ± SD (n = 3); error bars represent standard deviation.

Figure 8 presents the pore structure parameters measured by the Archimedes method and physical properties of ceramsite samples prepared from powders with different grinding times.

S1 has the highest total porosity at 28.74 ± 0.79%, the highest apparent porosity at 19.74 ± 0.77%, and the lowest closed porosity at 9.00 ± 0.40%. The coarse particles and low specific surface area cause poor combustion dispersion and low reactivity at high temperatures, severely limiting anorthite formation. The loose pore structure results in the highest water absorption at 10.56 ± 0.75% and the lowest compressive strength at 9.45 ± 0.44 MPa.

S2 shows a total porosity of 19.96 ± 0.69%, an apparent porosity of 6.28 ± 0.57%, and a closed porosity of 13.68 ± 0.52%. Combined with XRD semi-quantitative phase data, the low-open and high-closed pore structure of S2 is reasonably interpreted as a consequence of the concentrated heat release from 2 h milled powder, which may facilitate sufficient anorthite formation to fill intergranular voids in the mullite skeleton. Anorthite fills the interstices within the mullite skeleton, resulting in a water absorption of 2.99 ± 0.44% and a compressive strength of 15.12 ± 0.43 MPa.

S3 shows a total porosity of 22.10 ± 0.78%, an apparent porosity of 11.92 ± 0.76%, and a closed porosity of 10.19 ± 0.42%. S4 shows a further increase in total porosity at 23.04 ± 0.84% and apparent porosity at 14.41 ± 0.96%, and a further decrease in closed porosity at 8.62 ± 0.46%. These changes arise from the broadened particle size distribution (S3) and intensified agglomeration (S4), both of which disrupt combustion synchronicity and lead to a less uniform temperature distribution. The high-temperature reaction efficiency drops, anorthite formation is suppressed, and the pores inside agglomerates are not filled by the liquid phase. Consequently, water absorption increases to 5.83 ± 0.51% for S3 and 7.14 ± 0.62% for S4, while compressive strength decreases to 12.83 ± 0.49 MPa and 11.05 ± 0.54 MPa, respectively.

Statistical analysis via one-way ANOVA confirmed that the differences in compressive strength and water absorption among the four groups of ceramsite samples were statistically significant (*p* < 0.05).

### 3.5. Performance Regulation Mechanism

After 1 h of grinding, the powder remains coarse with limited specific surface area, resulting in the lowest reactivity and the poorest ceramsite performance among all samples.

Within 2 h of grinding, particles are continuously refined and the powder remains well dispersed. The concentrated particle size distribution allows sufficient contact between carbonaceous matter and oxygen, enabling low-temperature combustion to be completed within a narrow temperature range with the most intensive heat release. This concentrated heat release is inferred to form relatively uniform thermal fields during high-temperature sintering, which may enhance the reaction driving force and facilitate sufficient anorthite formation. Anorthite fills the gaps between mullite skeletons, reducing apparent porosity and increasing closed porosity, resulting in a high-strength ceramsite with water absorption of 2.99 ± 0.44% and compressive strength of 15.12 ± 0.43 MPa.

At 3 h of grinding, the broadened particle size distribution causes kinetic differences among particles of varying sizes, dispersing combustion heat release over a wider temperature range. At 4 h, signs of agglomeration appear to intensify, which may further disturb combustion synchronicity. Both mechanisms suppress anorthite formation and densification, leading to increased apparent porosity, decreased closed porosity, and deteriorated ceramsite performance.

To mitigate such over-grinding-induced agglomeration, future process optimization may consider introducing suitable anti-agglomeration additives. These additives are expected to suppress secondary coalescence of particles, thereby preserving a concentrated particle size distribution and combustion synchronicity. However, these additives may introduce potential side effects, such as alterations to sintering behavior, contamination risks, and increased costs, all of which warrant systematic evaluation prior to practical application.

The grinding parameters and ceramsite performance of this study are compared with those of other coal gangue-based ceramsite studies, as shown in Table 4.

Data are presented as mean ± SD based on triplicate measurements (n = 3). For references where SD was not reported in the original source, only the mean values are listed.

Most existing studies on coal gangue ceramsite control powder particle size by sieving after conventional grinding, and rarely focus on the evolution of particle size distribution during grinding and its intrinsic impact on ceramsite performance. The optimal sample in this study, sintered at 1200 °C after 2 h of ball milling, achieves a compressive strength of 15.12 ± 0.43 MPa and a water absorption of 2.99 ± 0.44%, which reaches the level of high-strength coal gangue-based ceramsite reported in related studies.

## 4. Conclusions

This study systematically investigated the effect of particle size distribution on the sintering behavior and properties of coal gangue ceramsite by controlling grinding time from 1 to 4 h. The main conclusions are as follows:

With increasing grinding time from 1 to 4 h, D50 continuously decreases, while the specific surface area increases to a peak at 3 h (2.286 m^2^/cm^3^) and then decreases at 4 h (2.193 m^2^/cm^3^) with little change in D50, which is commonly associated with fine-particle agglomeration. Within 2 h, particle refinement dominates; at 3 h, the particle size distribution broadens significantly; and from 3 to 4 h, signs of agglomeration become more apparent.

The powder ground for 2 h, with its concentrated particle size distribution, achieves more complete low-temperature combustion and more concentrated heat release. At 3 h, the broadened particle size distribution and the DSC curve both show shoulder peaks, indicating dispersed heat release. At 4 h, agglomeration further disperses heat release and reduces the extent of reaction.

The powder ground for 1 h is composed of coarse particles with low chemical reactivity, and the corresponding ceramsite delivers the poorest comprehensive performance among all samples. The concentrated heat release captured by TG-DSC for the 2 h ground powder is presumed to promote high-temperature mineral phase reconstruction and liquid phase formation, which accounts for the maximum anorthite content and a dense microstructure with low apparent porosity and abundant closed pores. Accordingly, the ceramsite prepared from 2 h ground powder achieves optimal performance for the Datong coal gangue, grinding parameters and sintering regime adopted in this study: water absorption of 2.99 ± 0.44% and compressive strength of 15.12 ± 0.43 MPa. In contrast, powders milled for 3 h and 4 h exhibit dispersed heat release during low-temperature combustion, which generates uneven thermal fields inside green pellets. Such thermal inconsistency inhibits mineral phase transformation and liquid phase generation, resulting in higher apparent porosity, lower closed porosity and deteriorated ceramsite properties.

The coal gangue ceramsite prepared in this study shows good application potential in lightweight concrete, thermal insulation building materials, road backfill and mine ecological restoration. In future work, we will further optimize the preparation process of ceramsite, test its long-term durability, and explore its practical applications in construction engineering and ecological restoration.

## Figures and Tables

**Table 1:** Main chemical composition of coal gangue raw materials (wt%).

Al_2_O_3_	SiO_2_	Fe_2_O_3_	Na_2_O	SO_3_	MgO	K_2_O	CaO	TiO_2_	LOI
40.04	22.89	4.18	0.44	1.10	2.53	2.02	4.80	1.04	20.18

LOI: loss on ignition.

**Table 2:** Industrial analysis and calorific value of coal gangue raw materials.

M_ad_ (%)	A_ad_ (%)	V_ad_ (%)	FC_ad_ (%)	Q_net,V,Mad_ (kcal/kg)
0.22	78.63	15.33	5.82	699.77

M_ad_—moisture on air-dry basis, %; A_ad_—ash on air-dry basis, %; V_ad_—volatile on air-dry basis, %; FC_ad_—fixed carbon on air-dry basis, %; Q_net,V,Mad_—low calorific value, kcal/kg.

**Table 3:** Particle size parameters of powders ground for different times.

Grinding Time (h)	D10 (μm)	D50 (μm)	D90 (μm)	Span	Volume Fraction by Particle Size Interval (vol%)			
					<2 μm	2–11 μm	11–22 μm	>22 μm
1	1.737	9.289	25.554	2.564	13.38	42.28	28.57	15.77
2	1.392	8.498	23.941	2.653	18.59	40.02	28.71	12.68
3	1.025	5.344	17.448	3.073	28.33	42.77	25.70	3.20
4	1.087	5.209	17.711	3.191	26.48	44.30	26.19	3.03

**Table 4:** Comparison of coal gangue ceramsite from representative studies with different grinding and sintering conditions.

Raw Materials	Grinding Condition & Particle Size	Compressive Strength (MPa)	Water Absorption (%)	Reference
Coal gangue (Datong, Shanxi)	Ball milling for 2 h; D_50_ = 8.498, specific surface area = 2.286 m^2^/cm^3^	15.12 ± 0.43	2.99 ± 0.44	This study
Coal gangue (Ordos, Inner Mongolia)	Ball milling to fine powder	-	17–20	[28]
Coal gangue (Ordos, Inner Mongolia)	Ball milling and sieving through 200 mesh (~75 μm)	5.0–12.9	0.5–9.9	[35]
Coal gangue and fly ash and steel slag	Grinding and passing through a 0.45 mm sieve	21.17	1.35	[40]
Coal gangue and iron ore tailings	Ball milling to <0.074 mm of 60 %	7.3	8.83	[41]
Coal gangue (CG) and iron ore tailings (IOT)	Specific surface areas of CG and IOT are 5010 and 3259 cm^2^/g	6.9	<10 %	[42]
Coal gangue (Anhui, China)	Specific surface areas = 5010 cm^2^/g	5.7	10.5	[42]

## Data Availability

The original contributions presented in this study are included in the article. Further inquiries can be directed to the corresponding author.
